# Comparison data of transcriptomes from blastocyst seeding samples and cultured cell lines from pigs

**DOI:** 10.1016/j.dib.2023.109212

**Published:** 2023-05-07

**Authors:** Jong-Nam Oh, Jinsol Jeong, Mingyun Lee, Gyung Cheol Choe, Kwang-Hwan Choi, Dong-Kyung Lee, Seung-Hun Kim, Chang-Kyu Lee

**Affiliations:** a Department of Agricultural Biotechnology, Animal Biotechnology Major, and Research Institute for Agriculture and Life Sciences, Seoul National University, Seoul 08826, Korea; b Designed Animal and Transplantation Research Institute (DATRI), Institute of Green Bio Science and Technology, Seoul National University, Pyeongchang 25354, Korea

**Keywords:** RNA-seq, Pig, Blastocyst, Epiblast-like cells, Primitive endoderm-like cells, Trophoblast-like cells, Mesoderm-like cells, Embryonic stem cell-like cells

## Abstract

Fertilized embryos develop and move freely in the reproductive tract until implantation. Subsequently, the embryos continue to develop after attachment to the uterus. Because of the absence of the uterus, in vitro culturing of embryos is limited to a period of approximately a week. Hatched blastocysts were seeded on feeder cells to extend the culture period. We cultured the colonies formed from the blastocysts for an additional 14 days. From the colonies, four types of cells were established, and each type was isolated to extract RNA. RNA sequencing was conducted using NovaSeq6000. Sequencing reads were aligned to genes and transcripts. Raw data from our previous study were used to compare these samples with the cultured cell lines. We analyzed differentially expressed genes and Gene Ontology terms between new samples and cultured cell lines. Our data can provide essential information for extending the period of embryo culture *in vitro*.

**Specifications Table**

**Table:** 

Subject	Bioinformatics
Specific subject area	RNA-seq of cultured cells from porcine blastocyst
Type of data	RNA sequencing data, Table, Figure
How the data were acquired	Samples were prepared as decribed below. Blastocysts seeding samples; Maturated oocytes were co-incubated with sperms for 4 h and *in vitro* cultured in 7 days. Blastocysts were attached on the feeder cells and colonies were cultured for 14 days. Four type of cells (type A, B, C, and D) were isolated from the colonies. Next generation sequencing using NovaSeq6000 and additional analysis. Adapters were applied on RNAs isolated from the samples. Sequencing was performed using HiSeq2500 (Illumina) Quality of the reads were checked and adapters were trimmed out. The transcriptome data were compared with data from our previous report (embryonic stem cells and somatic cells)
Data format	Raw data in FASTQ file, Filtered and Analysed
Description of data collection	Sequencing reads were quality checked. low quality reads and adapter sequences were filtered. RNAs were mapped to the reference genome of pigs (Sscrofa11.1, GCA_000003025.6). Expression levels of each RNA were normalized among samples. Gene ontology (GO) used for analysis of differentially expressed genes. RNAs were extracted from cultured cells and used to prepare RNA libraries. Sequencing reads were produced from RNA libraries using NovaSeq6000 (Illumina) and mRNAs were filtered from the whole reads. One from BL seeding experiment and one from our previous report [1] were compared to discover differentially expressed genes. The genes were categorized by Gene Ontology.
Data source location	Institution: Seoul National University City/Town/Region: Seoul Country: Korea
Data accessibility	Raw data files were uploaded to the following NCBI website. https://identifiers.org/geo/GSE189477[2] Raw data from our previous study were used to compare our samples with cultured cell lines (embryonic stem cells and somatic cells) https://identifiers.org/geo/GSE120031[3] Dataset files (Dataset 2–5) are located on Figshare [4]. https://doi.org/10.6084/m9.figshare.21791813.v1
Related research article	JN Oh, J Jeong, M Lee, GC Choe, DK Lee, KH Choi, SH Kim, CK Lee, Characterization of multitype colonies originating from porcine blastocysts produced *in vitro*, Front. Cell Dev. Biol. 12 September 2022. https://doi.org/10.3389/fcell.2022.918222

## Value of the Data

• Four types of cells were established from blastocyst seeding and transcriptome profiles of samples are presented on this article.
• We compared the data with our previous report. DEGs were analysed between blastocyst seeding samples and cultured cell lines.
• Our data will provide basal information for expanded culture of porcine embryos. Also, we suggested comparative data of the four cell types from blastocyst seeding and cultured cell lines of previous study.

## 1 Objective

Our previous paper showed that we could establish four types of cells from different embryonic lineages. Unlike the article, we compared blastocyst seeding samples with pluripotent cells and differentiated cells (embryonic stem cells and fetal fibroblast cells) in this paper.

## 2 Data Description

Sequencing data were generated using Novaseq6000. Raw data files were uploaded to the following NCBI website.

https://identifiers.org/geo/GSE189477 [2]

Raw data from our previous study were used to compare our samples with cultured cell lines (embryonic stem cells and somatic cells).

https://identifiers.org/geo/GSE120031 [3]

Dataset files (Dataset 2–5) are located on Figshare [4].

https://doi.org/10.6084/m9.figshare.21791813.v1

### 2.1 Sample preparation and RNA extraction

Blastocysts were generated using *in vitro* fertilization, as described in our previous study [5]. The embryos were seeded on the feeder cells (mouse fetal fibroblasts). Four cell types were isolated from the culture after colony formation, and RNA was isolated from each cell type following our previous report [6]. Dataset 1 includes sample and sequencing information. Extraction of total RNA was conducted using the Clear-S™ kit following the manufacturer's protocol (Invirustech, Korea).

### 2.2 Quality control and assessment of RNA expression

**Fig. 1:**
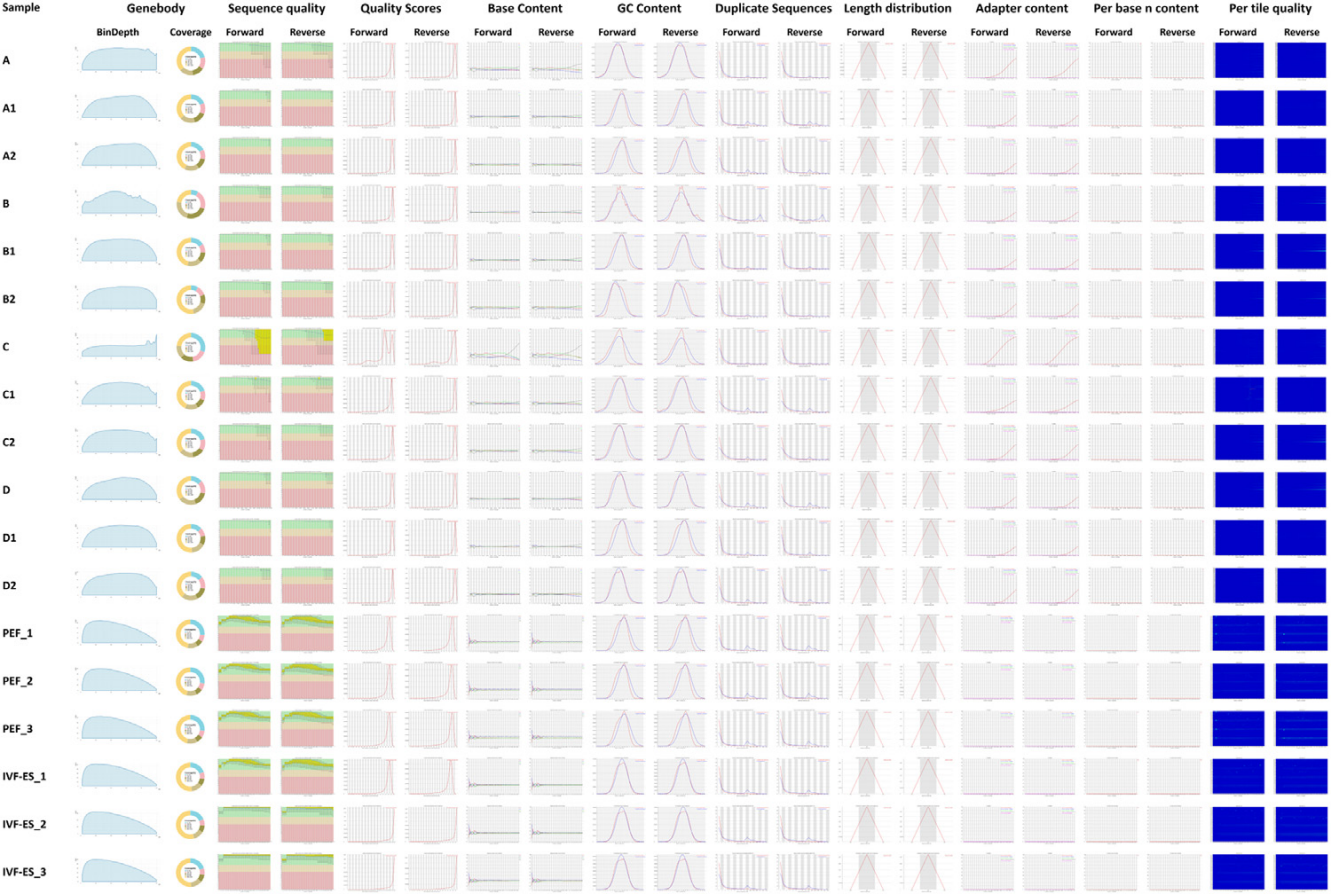
Quality control data of samples. BinDepth of Genebody shows degree of RNA degradation of sequencing reads. Coverage of Genebody shows coverage for protein coding genes in sequencing result. Sequence quality shows probability of error (Higher score means lower error rate of the read). Quality Scores means reliability of data. Base Count shows ratio and position of each base. GC content shows distribution of GC ratio within the reads. Duplicate Sequences shows copies of same read which is created during the sequencing procedure. Length distribution shows count of each length of bases. Adapter content shows position of adapter in reads. Per base N content (A sequencer has not sufficient confidence for a base, it shows the base as 'N'). Per tile quality shows whether there is loss in quality across the bases.

NovaSeq6000 (Illumina) was used for RNA sequencing. For the following analysis, raw data from sequencing (GSE189477) were used, whereas for comparative analysis, raw data from our previous reports (GSE120031) were used. Low-quality sequencing reads were filtered and the adaptor sequences in the remaining reads were trimmed off using Cutadapt. According to the FastQC test, the quality of the samples was sufficient for further analysis. (The data was shown in our previous report) [6]. We assessed the quality of the filtered reads through various criteria including BinDepth of Genebody (Fig. 1). Genes and transcripts were mapped, and their expression levels were normalized (Dataset 2). The data size, mapping ratio of each RNA-seq library, the correlations between biological replicates and the number of transcribed genes in each cell type were described in our previous publication [6].

### 2.3 Comparative analysis of RNAs

**Fig. 2:**
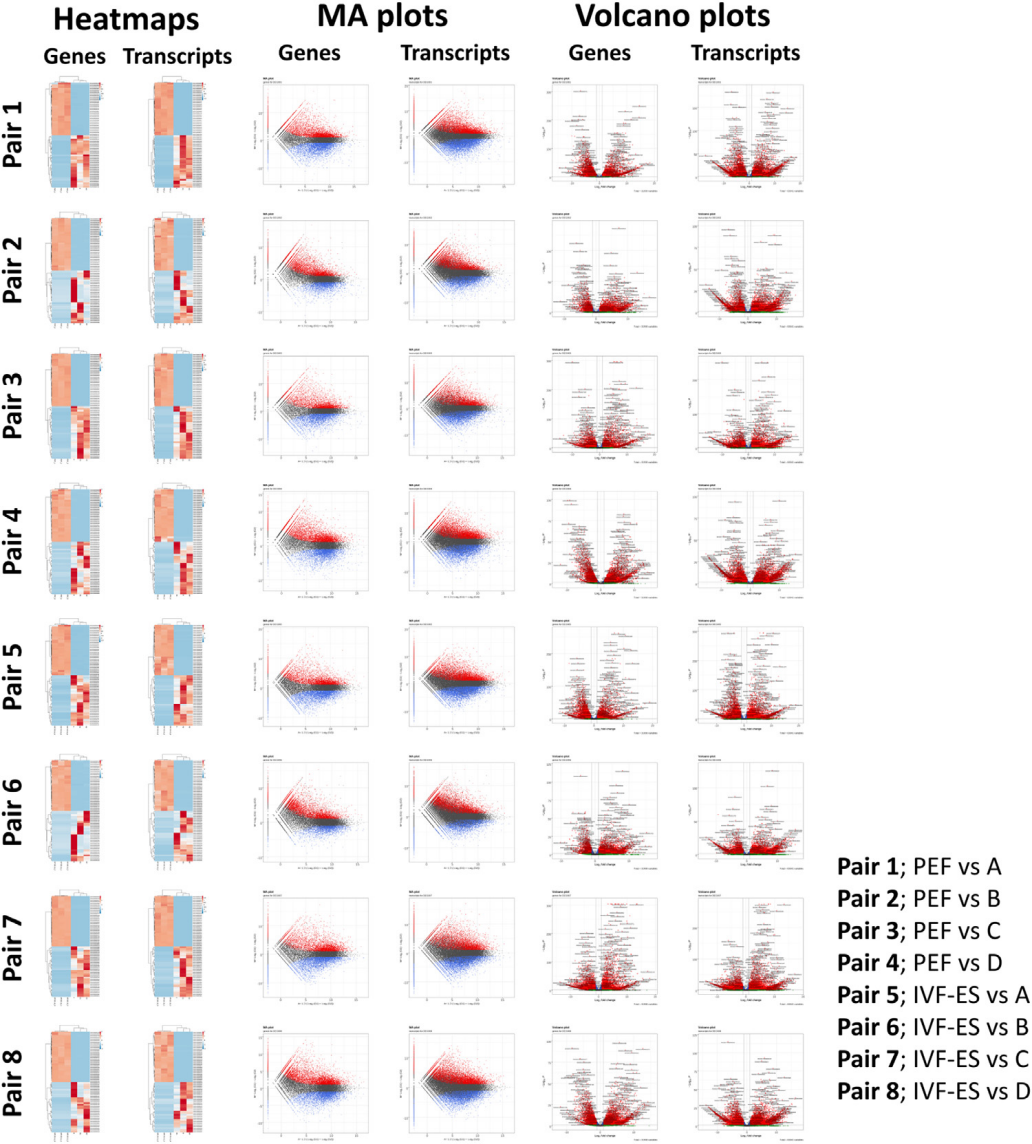
Heatmaps, MA plots, and volcano plots of differentially expressed genes. Heatmaps shows top 30 for up- and downregulated genes in each cell type. MA-plots and Volcano plots shows distribution of genes with 2D images. All the data were shown in the view of genes and transcriptions.

**Fig. 3:**
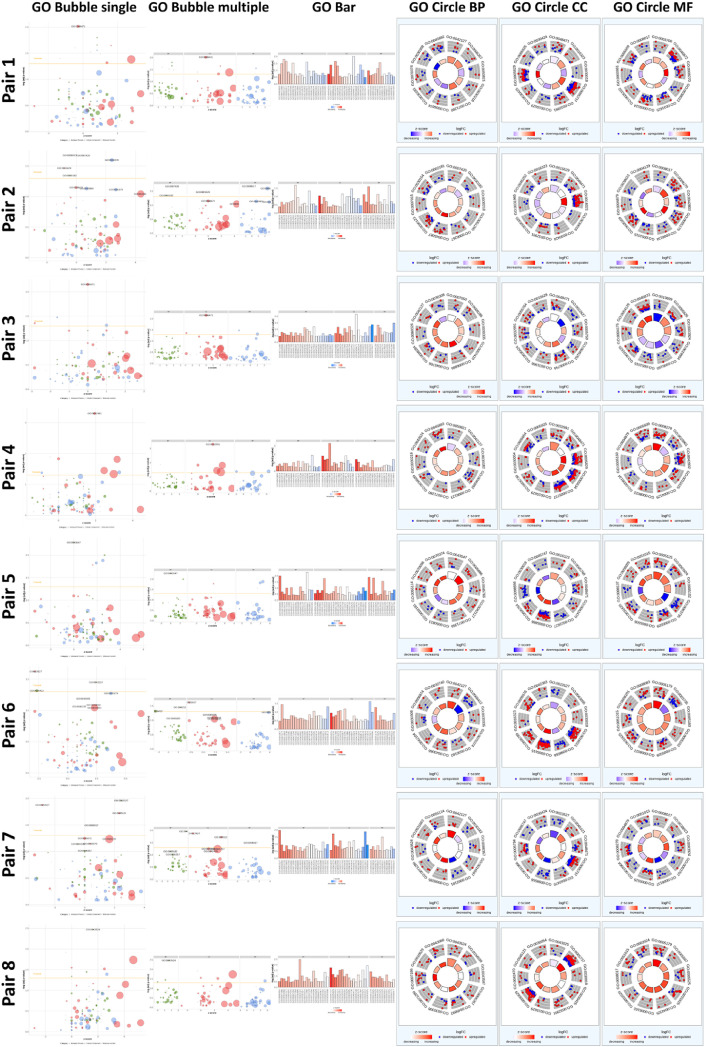
Gene Ontology (GO) analysis of differentially expressed genes. GO bubble plots show enrichment of GO terms. (Terms in Multiple are separated from Single) GO bar shows representative terms in each sample. GO Circles show top 10 terms which have the highest enrichment. BP; Biological process, CC; Cellular component, MM; Molecular function.

Data pairs for comparison are listed on sheet 1 of Dataset 3. Differentially expressed genes (DEGs) and their relative levels are organized in Dataset 3 and Fig. 2. Differential expression analysis was done based on the Negative Binomial (as known as Gamma-Poisson) distribution (*p* < 0.05 = significant difference). Volcano plots visualize distribution of DEGs (p-values and fold changes). MA plots show log2 fold changes (y-axis) and the mean of normalized counts (x-axis) on scatter plots. Dataset 4 contains the Gene Ontology (GO) terms for DEGs, which are depicted in Fig. 3.

## 3 Experimental Design, Materials and Methods

### 3.1 *In vitro* production of blastocyst and colony formation by blastocyst seeding

Blastocysts were produced using *in vitro* fertilization as described in our previous report [5]. The ovaries of prepubertal gilts were obtained from a local slaughterhouse and transferred to the laboratory in warmed saline. Cumulus–oocyte complexes (COCs) were obtained by aspirating 3- to 7-mm follicles of prepubertal gilts using a 10-ml syringe and an 18-gage needle. COCs with compact multiple layers of cumulus cells and fine cytoplasm were collected from aspirated porcine follicular fluid (pFF) and cultured for 44 h at 39 °C in tissue culture medium 199 (TCM 199; Gibco, Grand Island, NY, USA) supplemented with 10% pFF, l-cysteine (0.1 mg/ml), sodium pyruvate (44 ng/ml), epidermal growth factor (10 ng/ml), insulin (1 mg/ml), and kanamycin (75 µg/ml). The COCs were matured using 10 IU/ml gonadotropin hormones, pregnant mare serum gonadotropin (Lee Biosolutions, Maryland Heights, MO, USA), and human chorionic gonadotropin for the first 22 h. After maturation, cumulus cells were isolated from the oocytes using hyaluronidase. Sperm cells were washed two times with Dulbecco's phosphate-buffered saline supplemented with 0.1% bovine serum albumin (BSA) at 1400 rpm for 3 min. Washed sperm (4 × 10^4^/ml in final concentration) were then coincubated with matured oocytes in 500-µl modified Tris-buffered medium (mTBM) for 4 h (Abeydeera and Day, 1997). mTBM comprised 113.1-mM sodium chloride, 3-mM potassium chloride, 7.5-mM calcium chloride, 20-mM Trizma® base, 11-mM glucose, 5-mM pyruvate, 1-mM caffeine, and 0.8% BSA. After this process, the eggs were incubated in 5% CO_2_ and 5% O_2_ at 39 °C in 20 µl of porcine zygote medium 3. Hatched blastocysts (on day 7 after fertilization) were attached to the feeder cells (mitomycin C-treated mouse embryonic fibroblasts). The basal medium contained DMEM/F-12 supplemented with MEM non-essential amino acid, glutamax, 2-mercaptoethanol, antibiotic-antimycotic solution, and 15% KnockOut™ serum replacement (Gibco, NY, USA). Basic fibroblast growth factor (FGF) and human Leukemia Inhibitory Factor (LIF) were added to the medium (10 ng/ml each).

### 3.2 RNA isolation and sequencing

Total RNA was isolated from the samples using the Clear-S™ kit following the manufacturer's instructions (Invirustech, Korea). After validating the extraction (RNA concentration, optical density ratio, 28S:18S ratio, etc.), we prepared libraries using the SMART-Seq® v4 Ultra® Low Input RNA Kit for sequencing (Takara Bio, CA, USA), followed by RNA sequencing using NovaSeq6000 (Illumina).

### 3.3 Analysis of sequencing data

All program-based analyses were conducted using LifeGenomics, and the corresponding software and parameters are described in our previous article. Following the sequencing quality check, low-quality reads were filtered, and adapters were trimmed out. All the RNA reads were aligned to the reference genome (Sus scrofa 11.1, GCA_000003025.6).

### 3.4 Comparison of sequencing data with the previous report

Sequencing data of each cell type was paired with those of our previous report [7]. DEGs were identified and their relative levels were estimated (Dataset 3). Heatmaps depict the top 30 up- and downregulated genes in each comparison, and MA and volcano plots were created using DEGs (Fig. 2). GO terms were analyzed from DEG data (Dataset 4) and were classified into three categories—biological process, cellular component, molecular function—and visualized as depicted in Fig. 3. In this paper, we suggested a comparison between one of our new samples and one of our previous data (Pair 1 to 8). A list of samples is described in Table 1. Type A (epiblast-like) and type C (trophectoderm-like) cells are grown in monolayer, and type B (primitive endoderm-like) and type D (mesoderm-like) cells are grown in multilayer. Among four types, only type A and D are positive for AP staining (Fig. 3 of [6]). Up- or down-regulated genes within four types (A, B, C, and D) are listed in our previous paper [6].

**Table 1:** List of samples.

Access number of SRA	Access number of samples	Title of sample	Description	Sample name in this study
SRP347590[8]	GSM5702418	RNA-Seq of sus scrofa: cell population from blastocyst seeding [Type_A]	Epiblast-like cells	A
	GSM5702419	RNA-Seq of sus scrofa: cell population from blastocyst seeding [Type_A1]	Epiblast-like cells	A1
	GSM5702420	RNA-Seq of sus scrofa: cell population from blastocyst seeding [Type_A2]	Epiblast-like cells	A2
	GSM5702421	RNA-Seq of sus scrofa: cell population from blastocyst seeding [Type_B]	Primitive endoderm-like cells	B
	GSM5702422	RNA-Seq of sus scrofa: cell population from blastocyst seeding [Type_B1]	Primitive endoderm-like cells	B1
	GSM5702423	RNA-Seq of sus scrofa: cell population from blastocyst seeding [Type_B2]	Primitive endoderm-like cells	B2
	GSM5702424	RNA-Seq of sus scrofa: cell population from blastocyst seeding [Type_C]	Trophectoderm-like cells	C
	GSM5702425	RNA-Seq of sus scrofa: cell population from blastocyst seeding [Type_C1]	Trophectoderm-like cells	C1
	GSM5702426	RNA-Seq of sus scrofa: cell population from blastocyst seeding [Type_C2]	Trophectoderm-like cells	C2
	GSM5702427	RNA-Seq of sus scrofa: cell population from blastocyst seeding [Type_D]	Mesoderm-like cells	D
	GSM5702428	RNA-Seq of sus scrofa: cell population from blastocyst seeding [Type_D1]	Mesoderm-like cells	D1
	GSM5702429	RNA-Seq of sus scrofa: cell population from blastocyst seeding [Type_D2]	Mesoderm-like cells	D2

SRP161888[3]	GSM3391893	Somatic cell control (Rep.1)	Pig fetal fibroblasts	PEF_1
	GSM3391894	Somatic cell control (Rep.2)	Pig fetal fibroblasts	PEF_2
	GSM3391895	Somatic cell control (Rep.3)	Pig fetal fibroblasts	PEF_3
	GSM3391902	IVF-derived ESC-11 (Rep.1)	Pig ESCs	IVF-ES_1
	GSM3391903	IVF-derived ESC-11 (Rep.2)	Pig ESCs	IVF-ES_2
	GSM3391904	IVF-derived ESC-11 (Rep.3)	Pig ESCs	IVF-ES_3

### 3.5 Code availability and URLs of tools

No custom code used to process data in this study.

EnhancedVolcano - “EnhancedVolcano: publication-ready volcano plots with enhanced colouring and labeling” (https://bioconductor.org/packages/devel/bioc/vignettes/EnhancedVolcano/inst/doc/EnhancedVolcano.html, 2022).

FastQC - Andrews, S. “FastQC: A Quality Control Tool for High Throughput Sequence Data.” (https://www.bioinformatics.babraham.ac.uk/projects/fastqc, 2010).

PCAtools - “PCAtools: everything Principal Component Analysis” (https://bioconductor.org/packages/devel/bioc/vignettes/PCAtools/inst/doc/PCAtools.html, 2022).

## Ethics Statements

All the experiments were performed following the guidelines of the Institute of Laboratory Animal Resources, Seoul National University (SNU-140,328–2).

## CRediT authorship contribution statement

**Jong-Nam Oh:** Conceptualization, Methodology, Software, Formal analysis, Investigation, Data curation, Writing – original draft, Visualization. **Jinsol Jeong:** Methodology, Investigation, Resources. **Mingyun Lee:** Resources. **Gyung Cheol Choe:** Resources. **Kwang-Hwan Choi:** Conceptualization, Investigation, Writing – review & editing. **Dong-Kyung Lee:** Investigation, Resources. **Seung-Hun Kim:** Resources. **Chang-Kyu Lee:** Conceptualization, Supervision, Funding acquisition.

## Declaration of Competing Interest

The authors declare that they have no known competing financial interests or personal relationships that could have appeared to influence the work reported in this paper.

## Data Availability

Supplementary File for Comparison data of transcriptomes from blastocyst seeding samples and cultured cell lines from pigs (Original data) (Figshare). RNA-seq for cell populations from blastocyst seeding in pig (Reference data) (NCBI). Derivation of authentic porcine embryonic stem cells using defined culture conditions (Reference data) (NCBI).

## Appendix Supplementary materials

Image, application 1

Image, application 2

Image, application 3

Image, application 4

Image, application 5

Image, application 6
