# Supplementary material for: Comparison data of transcriptomes from blastocyst seeding samples and cultured cell lines from pigs
Source: Data Brief. 2023 May 7;48:109212. doi: 10.1016/j.dib.2023.109212 (PMC10196955; doi:10.1016/j.dib.2023.109212)
Supplement: Supplementary file 2 [file mmc2.pdf]

Figure 1-1. Quality control data of samples.

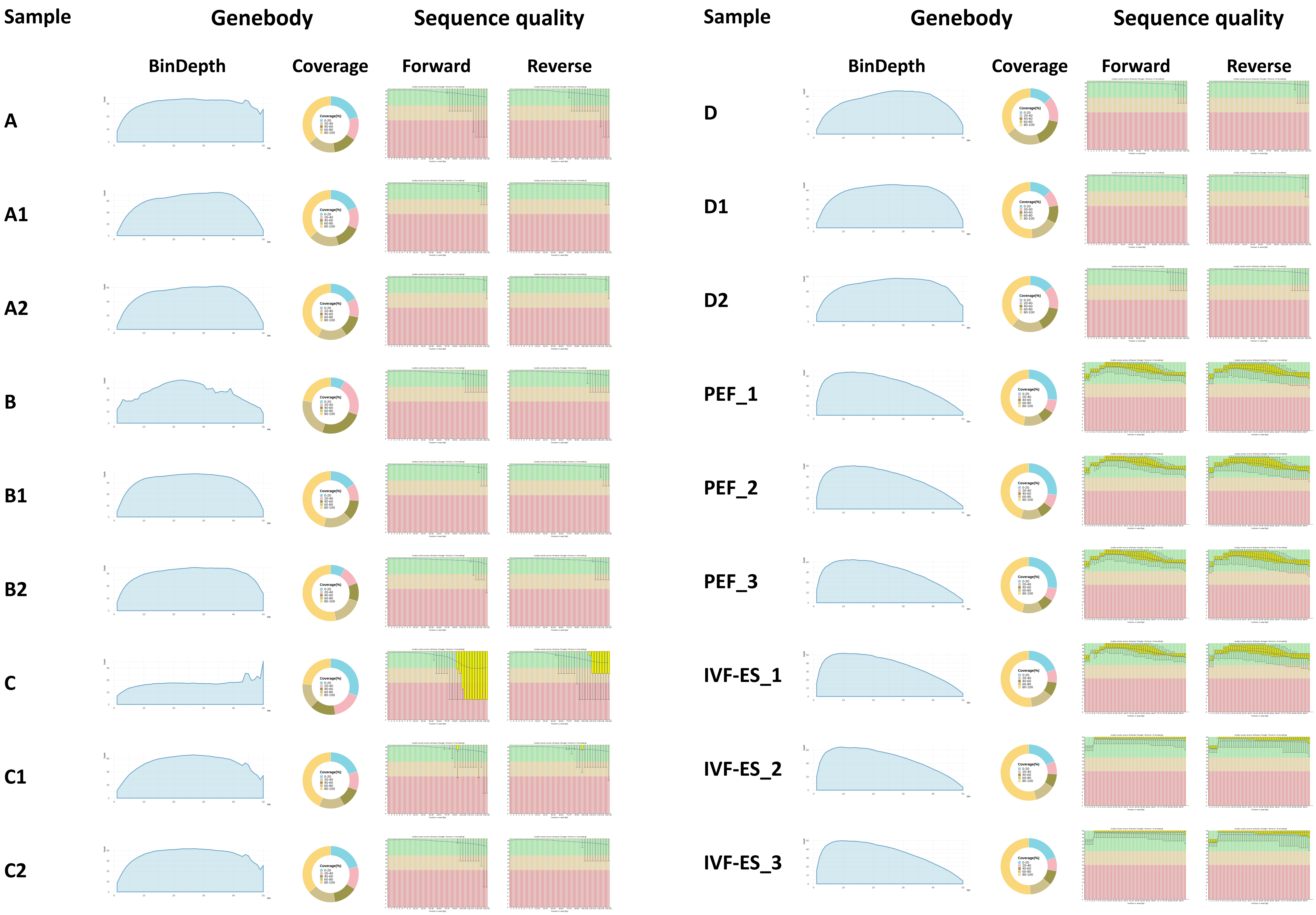

Figure 1-2. Quality control data of samples.

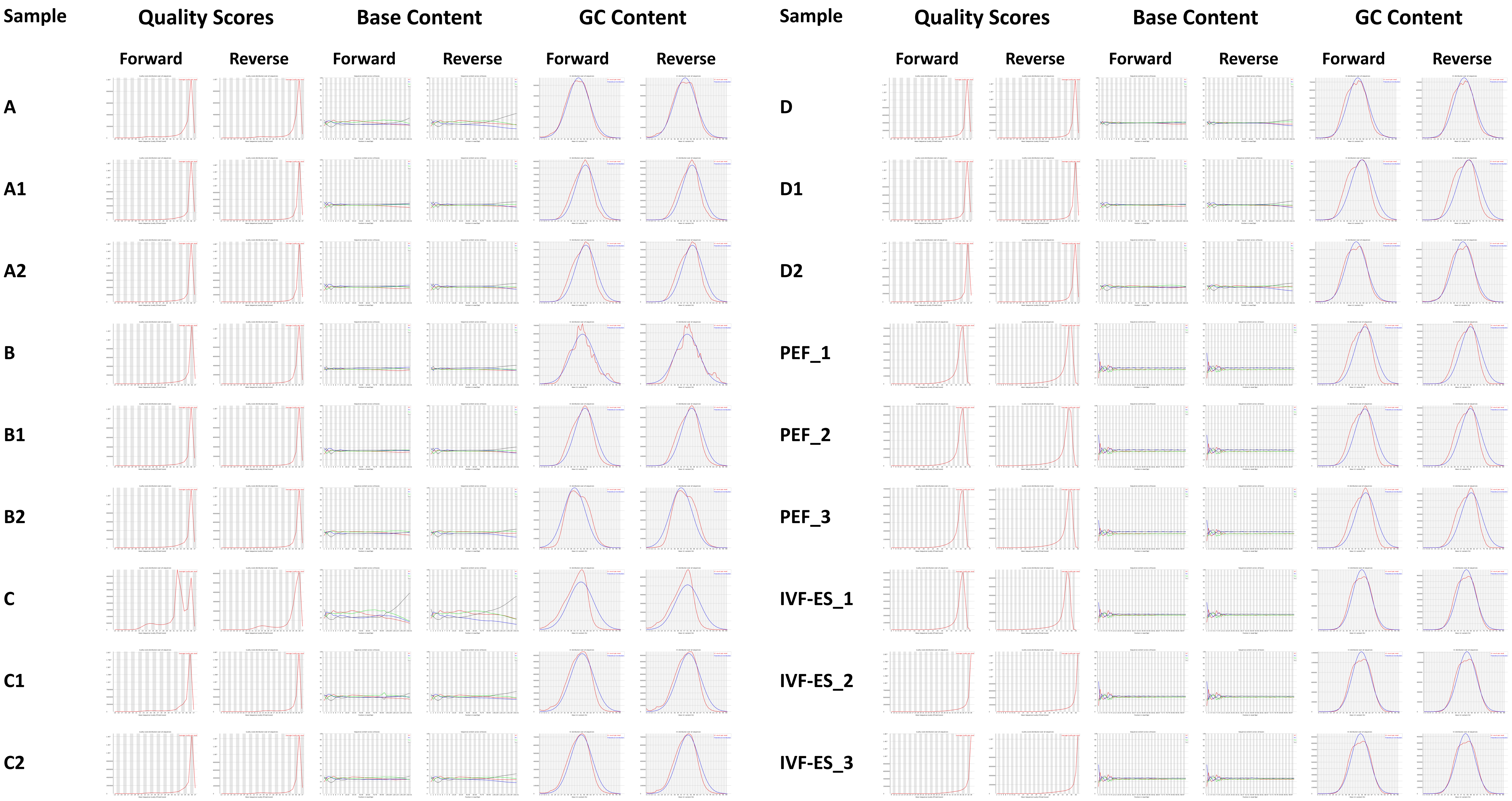

Figure 1-3. Quality control data of samples.

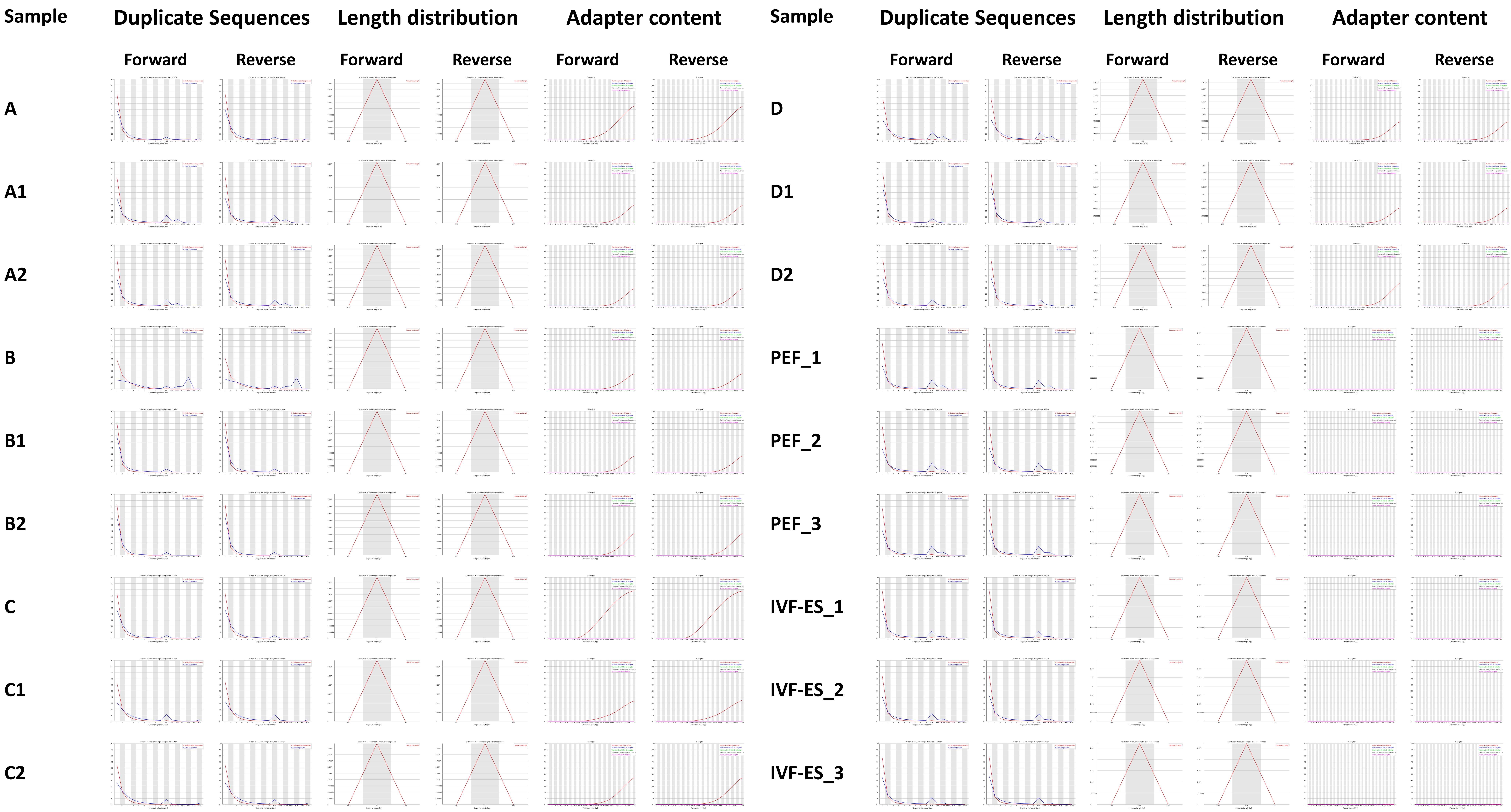

Figure 1-4. Quality control data of samples.

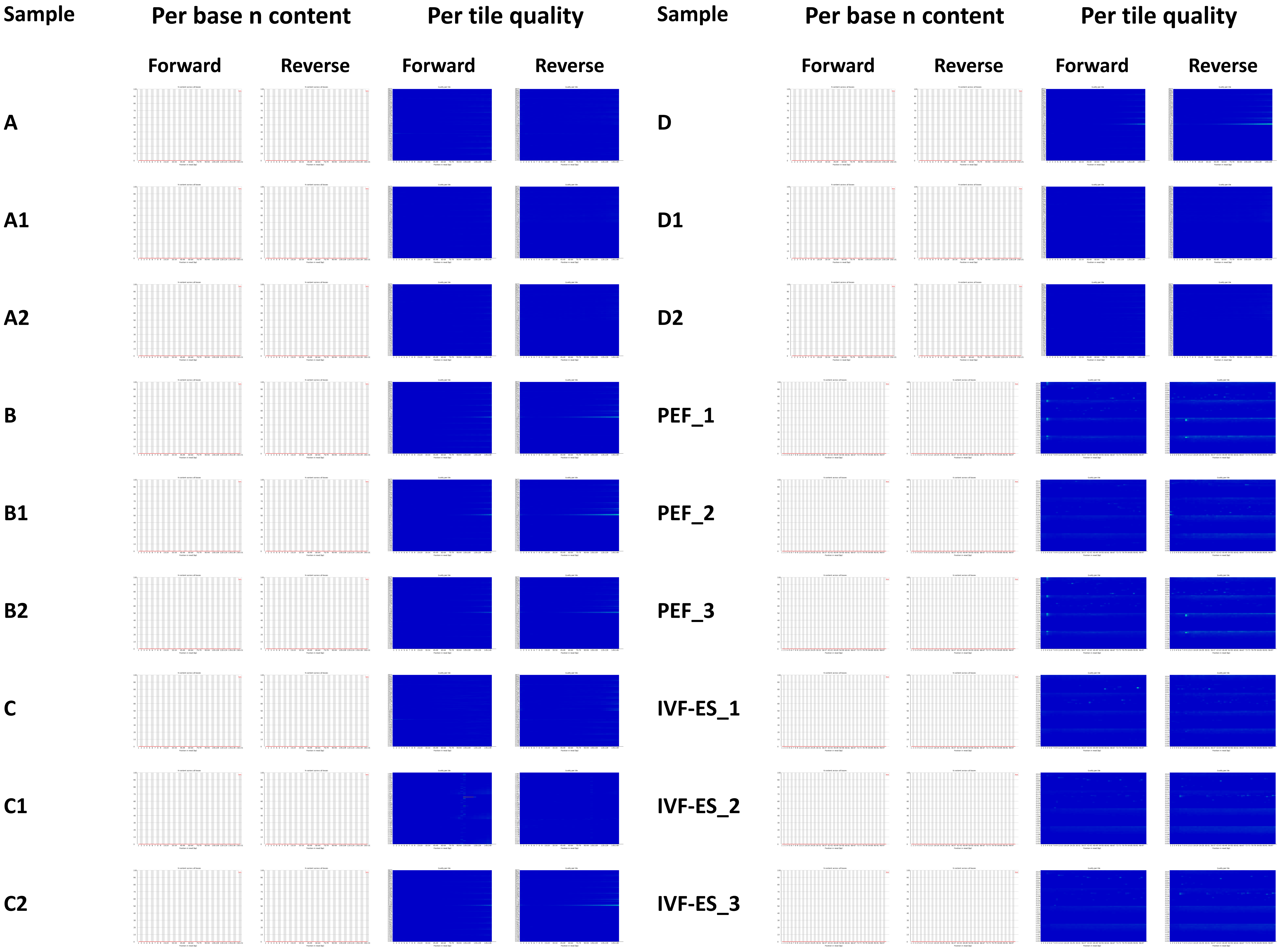

Figure 2-1. Heatmaps, MA plots, volcano plots of differentially expressed genes.

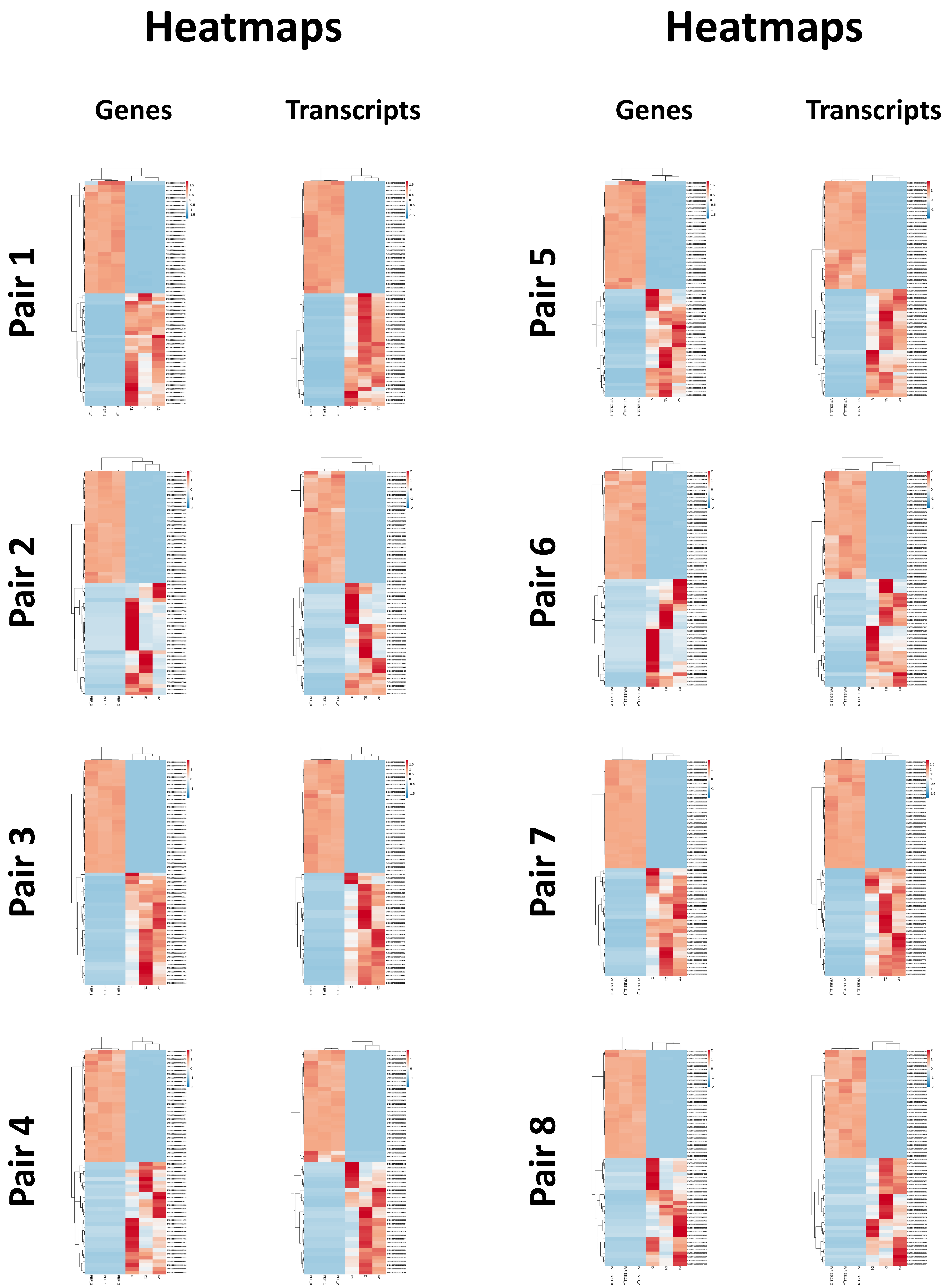

Pair 1; PEF vs A
Pair 2; PEF vs B
Pair 3; PEF vs C
Pair 4; PEF vs D
Pair 5; IVF-ES vs A
Pair 6; IVF-ES vs B
Pair 7; IVF-ES vs C
Pair 8; IVF-ES vs D

Figure 2-2. Heatmaps, MA plots, volcano plots of differentially expressed genes.

MA plots

Genes

Transcripts

Pair 1

Pair 2

Pair 3

Pair 4

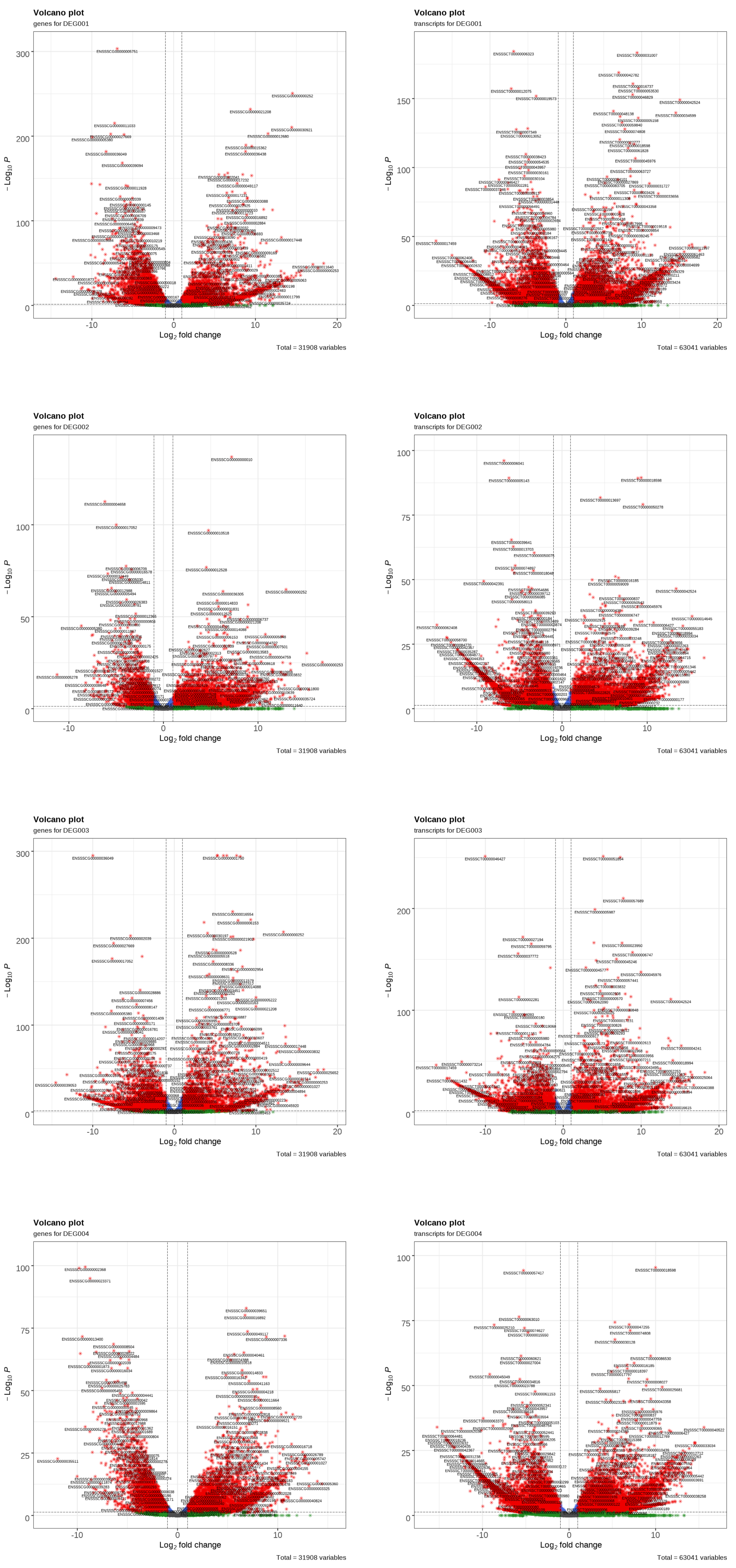

MA plots

Genes

Transcripts

Pair 5

Pair 6

Pair 7

Pair 8

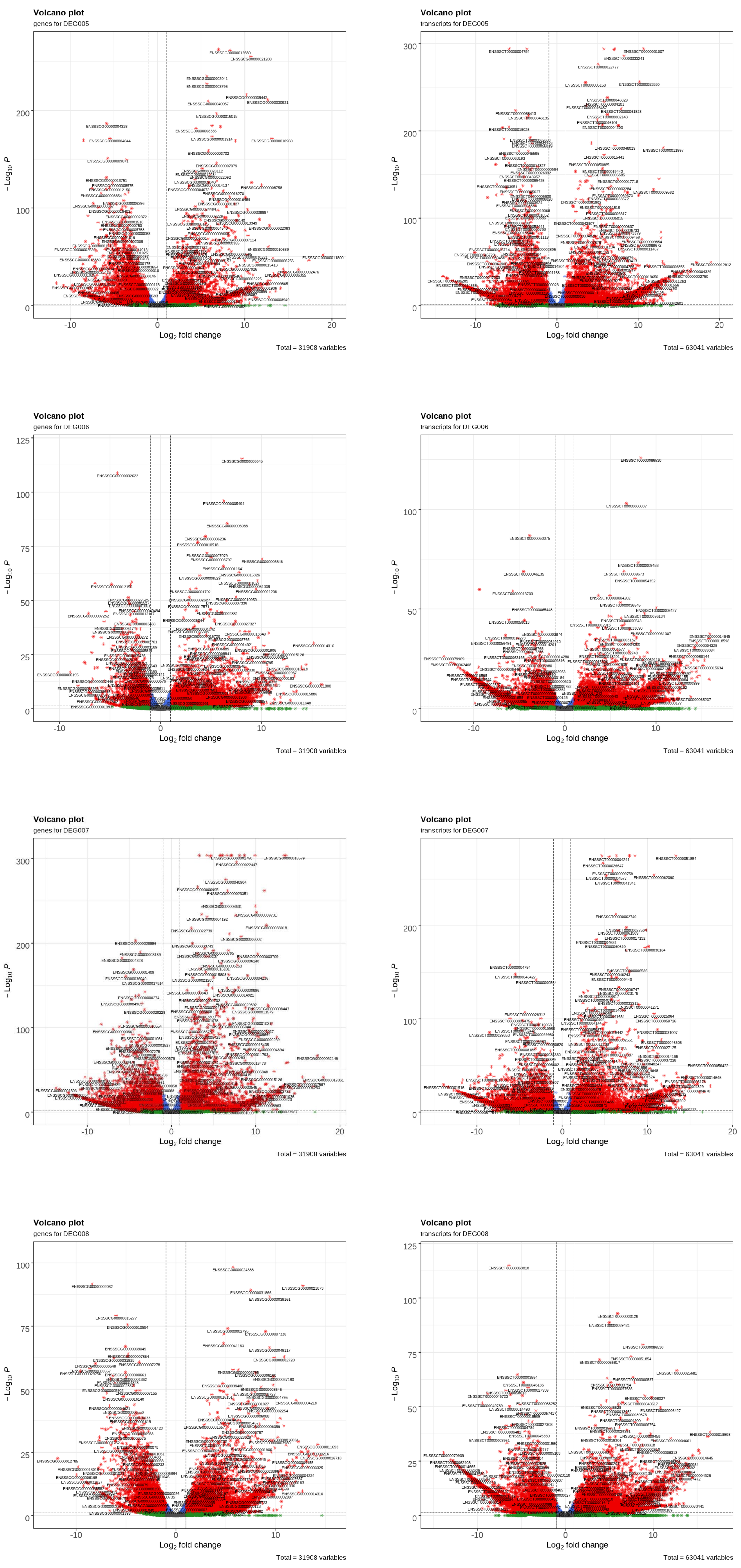

Pair 1; PEF vs A
Pair 2; PEF vs B
Pair 3; PEF vs C
Pair 4; PEF vs D
Pair 5; IVF-ES vs A
Pair 6; IVF-ES vs B
Pair 7; IVF-ES vs C
Pair 8; IVF-ES vs D

Figure 2-3. Heatmaps, MA plots, volcano plots of differentially expressed genes.

Volcano plots | Volcano plots

Genes | Transcripts | Genes | Transcripts

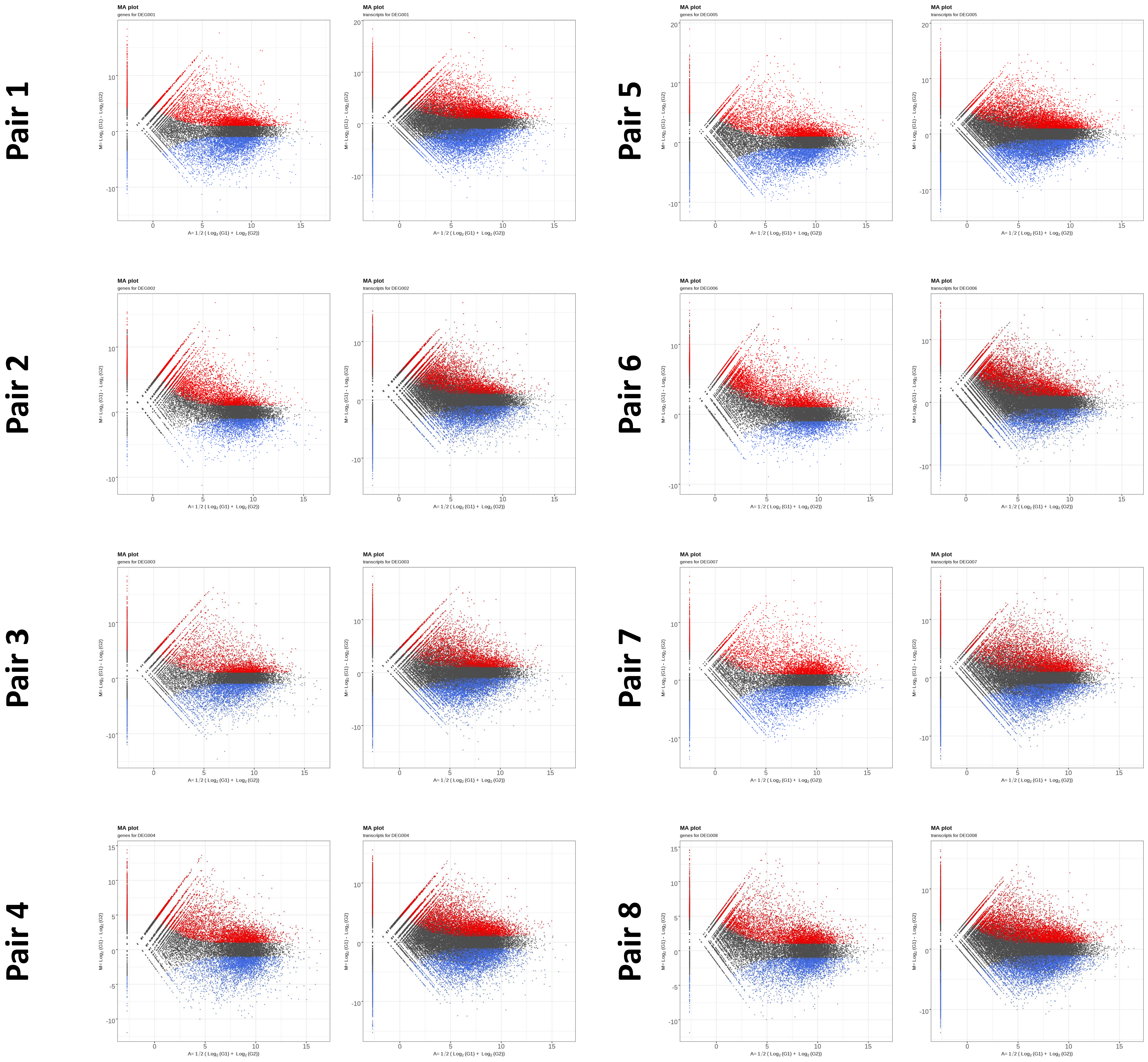

Pair 1; PEF vs A
Pair 2; PEF vs B
Pair 3; PEF vs C
Pair 4; PEF vs D
Pair 5; IVF-ES vs A
Pair 6; IVF-ES vs B
Pair 7; IVF-ES vs C
Pair 8; IVF-ES vs D

Figure 3-1. GO analysis of differentially expressed genes.

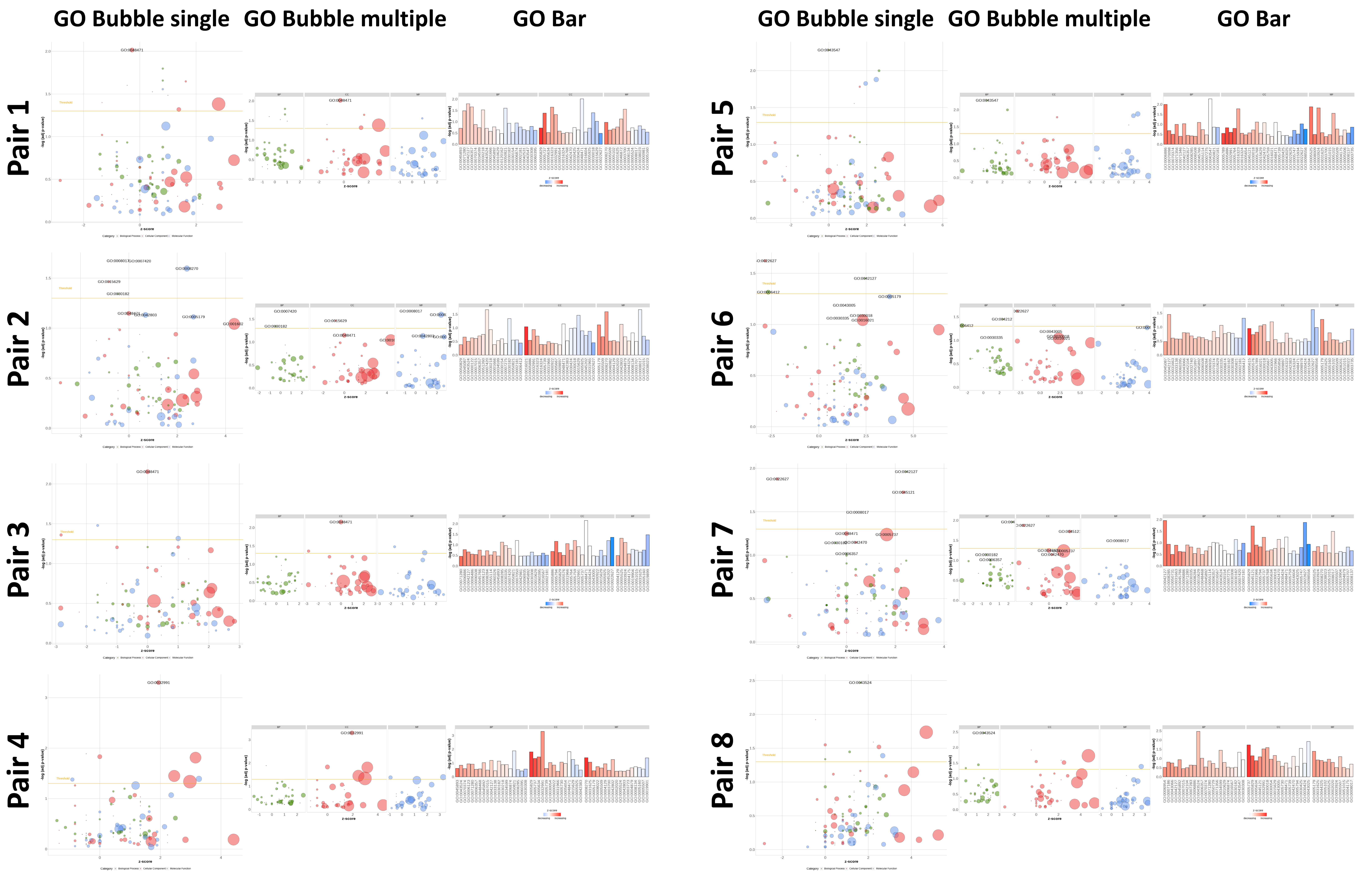

Pair 1; PEF vs A
Pair 2; PEF vs B
Pair 3; PEF vs C
Pair 4; PEF vs D
Pair 5; IVF-ES vs A
Pair 6; IVF-ES vs B
Pair 7; IVF-ES vs C
Pair 8; IVF-ES vs D

Figure 3-2. GO analysis of differentially expressed genes.

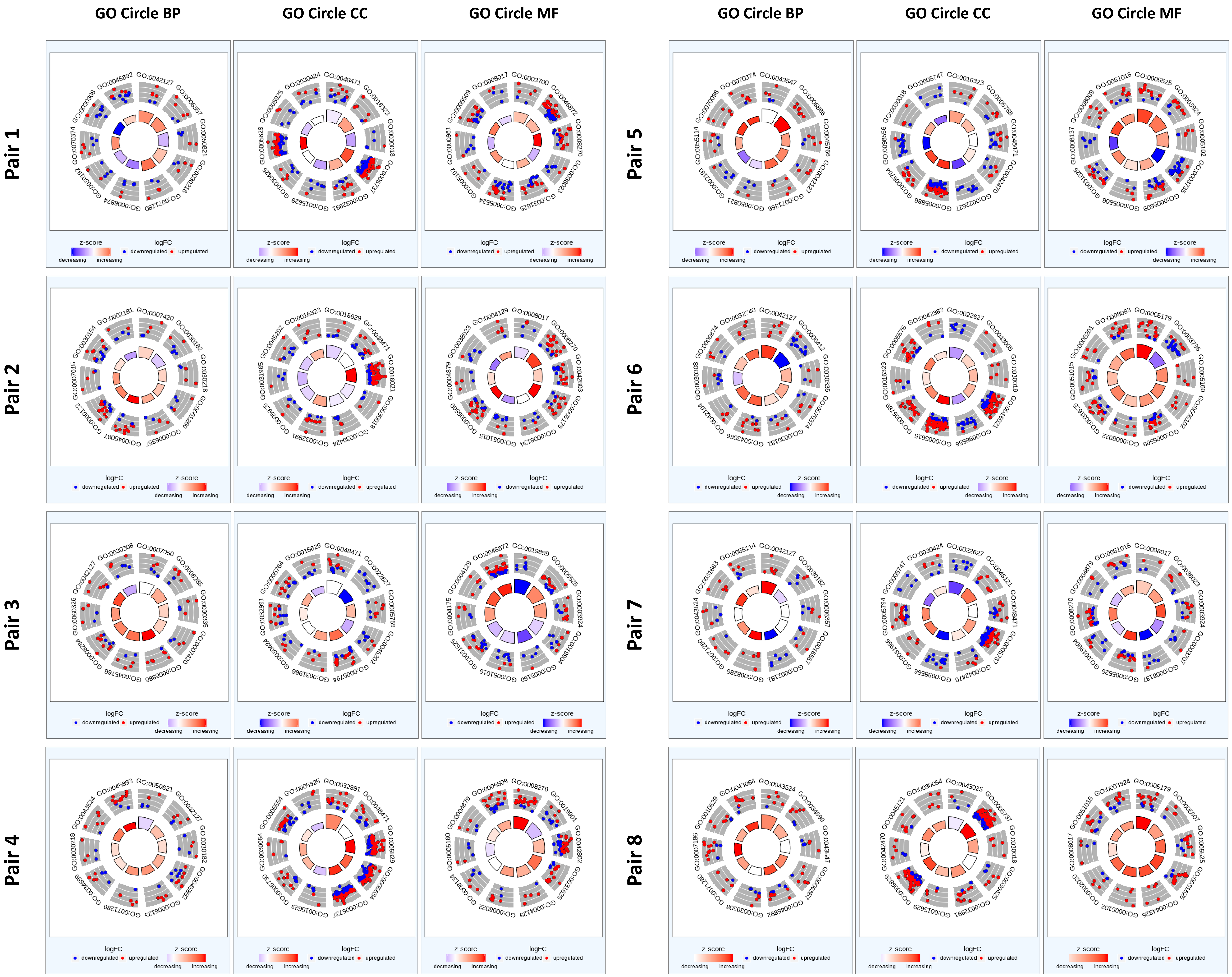

BP; Biological process
CC; Cellular component
MM; Molecular function
